# Adequacy of Clinical Information in Knee Radiography Requests With Reference to the Pittsburgh Knee Rule: A Single-Centre Clinical Audit

**DOI:** 10.7759/cureus.116695

**Published:** 2026-09-21

**Authors:** Paras Shrestha, Jae Yong Park

**Affiliations:** 1 Radiology, Dartford and Gravesham NHS Trust, London, GBR; 2 Medicine, Dartford and Gravesham NHS Trust, London, GBR

**Keywords:** clinical audit, clinical decision rule, diagnostic imaging, emergency medicine, fracture detection, healthcare costs, knee radiography, pittsburgh knee rule

## Abstract

Background and objective

Knee radiographs are frequently requested following acute knee injury, although the proportion demonstrating a fracture is relatively small. The Pittsburgh Knee Rule (PKR) is a validated clinical decision rule developed to support selective radiography following a fall or blunt knee trauma. Assessment of the rule, however, depends on adequate documentation of the relevant clinical findings. This clinical audit aimed to assess the adequacy of PKR-related clinical information documented on knee radiograph requests at a district general hospital with reference to the Royal College of Radiologists (RCR) AuditLive standard. Secondary objectives were to determine fracture yield and identify areas for improvement in request documentation.

Materials and methods

A single-centre retrospective clinical audit was undertaken. A total of 300 knee radiograph records were initially identified from two source lists. Ninety-five records were excluded during initial eligibility screening because they fell outside the predefined audit population, and three repeated or duplicate records were subsequently removed during dataset consolidation, resulting in 202 requests for analysis. The original radiology requests and corresponding imaging reports were reviewed for patient age, documented mechanism of injury, weight-bearing information, and radiographic evidence of acute fracture. The PKR was considered applicable where a fall or blunt-trauma mechanism was documented. Requests were classified as documented PKR-positive, documented PKR-negative, indeterminate because of insufficient documentation, or PKR not applicable where the documented mechanism fell outside the intended scope of the rule.

Results

Of 202 knee radiograph requests, 83 (41.1%) were classified as documented PKR-positive, 78 (38.6%) as indeterminate because of insufficient documentation, and 41 (20.3%) as PKR not applicable. No request contained sufficient information to establish a confidently documented PKR-negative status. Acute fractures were identified in 19/202 (9.4%) examinations. Fractures occurred in 15/83 (18.1%) documented PKR-positive requests and 4/78 (5.1%) requests with indeterminate documentation. No fractures occurred among the 41 requests classified as PKR not applicable.

Conclusions

A substantial proportion of knee radiograph requests lacked sufficient documented clinical information to permit reliable assessment with reference to the PKR. These findings reflect information available within radiology requests rather than confirmed real-time application of the rule by requesting clinicians. Absence of a documented criterion should not be interpreted as evidence that the corresponding clinical finding was absent or that radiography was inappropriate. An educational intervention was subsequently introduced, with a second audit cycle planned to assess improvement in documentation.

## Introduction

Knee radiographs are frequently requested following acute trauma, although the proportion demonstrating a fracture is relatively small. Excessive imaging may contribute to increased patient waiting times, radiology workload, healthcare expenditure, and unnecessary exposure to ionising radiation. Previous UK data have demonstrated high rates of radiography among patients presenting with acute knee injuries despite a comparatively low fracture yield [1].

The Pittsburgh Knee Rule (PKR) was developed to support more selective use of radiography following a fall or blunt knee trauma. For patients with an applicable mechanism, radiography is recommended where the patient is younger than 12 years, older than 50 years, or unable to walk four weight-bearing steps [2]. The original validation study reported a sensitivity of 100% and a specificity of 79% [2], while subsequent multicentre prospective evaluations demonstrated similarly high sensitivity [3].

Application of the PKR depends on the relevant clinical information being available. In routine radiology practice, the information available to the radiology service is primarily that provided in the imaging request. The Royal College of Radiologists (RCR) AuditLive standard therefore recommends that relevant requests for knee radiography contain sufficient clinical information to permit assessment with reference to the PKR [4].

This clinical audit aimed to assess the adequacy of PKR-related clinical information documented on knee radiograph requests, rather than to determine whether clinicians prospectively applied the rule during real-time clinical assessment. Secondary objectives were to determine fracture yield, identify deficiencies in request documentation, and use the findings to inform a targeted educational intervention.

## Materials and methods

A single-centre retrospective clinical audit was conducted at a district general hospital to assess the adequacy of clinical information documented on knee radiograph requests with reference to the PKR and the RCR AuditLive standard [4].

Patient selection

A total of 300 knee radiograph records were initially identified from two source lists. During initial eligibility screening, 95 records were excluded because they fell outside the predefined audit population. Exclusion criteria included chronic or non-traumatic knee symptoms, examinations performed primarily for suspected degenerative disease, postoperative or longer-term follow-up examinations, and other presentations unrelated to the acute knee-injury cohort.

Three repeated or duplicate records were subsequently removed during consolidation of the screened datasets, resulting in a final audit cohort of 202 knee radiograph requests.

The historical screening record identified which examinations were excluded but did not consistently preserve a discrete exclusion reason for every excluded record. Consequently, reliable numerical subdivision of all 95 screening exclusions according to individual exclusion criterion was not possible retrospectively.

Data collection

For each included examination, the original radiology request and corresponding imaging report were reviewed. Data collected included patient age, documented mechanism of injury, documentation relating to weight-bearing ability, and radiographic evidence of acute fracture.

The audit assessed information recorded within the radiology request. It could not establish whether additional clinical findings had been assessed by the requesting clinician but had not been documented.

Documentation-based PKR classification

The original PKR was developed for patients presenting following a fall or blunt trauma [2,3]. The documented mechanism of injury was therefore considered before applying the age and weight-bearing components of the rule.

A request was classified as documented PKR-positive where a fall or blunt-trauma mechanism was documented together with at least one positive criterion: age younger than 12 years, age older than 50 years, or documented inability to walk four weight-bearing steps.

A request could only be classified as documented PKR-negative where all of the following were documented: a PKR-applicable mechanism, age between 12 and 50 years, and explicit ability to walk four weight-bearing steps.

Where a potentially applicable request lacked one or more of the clinical elements required to determine PKR status, it was classified as indeterminate. An undocumented clinical finding was therefore not assumed to be absent.

Requests documenting an isolated twisting, hyperextension, non-contact, or other mechanism outside a fall or blunt trauma were classified as PKR not applicable, rather than PKR-negative.

The PKR was applied as originally described by Seaberg and colleagues [2,3]. No copyrighted figures, tables, questionnaires, or other proprietary materials were reproduced or adapted; therefore, separate permission for reproduction was not required.

Outcomes and analysis

The primary outcome was the adequacy of documented clinical information to permit assessment with reference to the PKR and the RCR AuditLive standard [4]. Secondary outcomes included overall fracture yield and fracture distribution across the documentation-based PKR categories.

Data were analysed descriptively and anonymised prior to analysis. Sensitivity, specificity, positive predictive value, and negative predictive value were not calculated in the revised analysis. Re-review demonstrated that no request contained sufficient information to establish a confidently documented PKR-negative cohort. Diagnostic-performance estimates based on the original binary classification would therefore have required undocumented clinical findings to be treated as absent and were considered potentially misleading.

Educational intervention

Following completion of the first audit cycle, an educational poster summarising the PKR and the clinical information required for the assessment of knee radiograph requests was displayed in the Accident and Emergency department.

The intervention emphasised documentation of mechanism of injury, relevant age criteria, and ability to walk four weight-bearing steps. A second audit cycle was planned to assess whether this intervention improved request documentation and compliance with the RCR standard.

## Results

Patient cohort and documentation-based classification

A total of 202 knee radiograph requests were included in the final audit cohort (Table 1). Following re-review using the documentation-based classification, 83/202 (41.1%) requests were classified as documented PKR-positive. A further 78/202 (38.6%) were classified as indeterminate because the clinical information available within the request was insufficient to establish PKR status. Forty-one of 202 (20.3%) requests involved mechanisms outside a fall or blunt trauma and were therefore classified as PKR not applicable. No request contained sufficient documented information to establish a confidently documented PKR-negative status.

**Table 1:** Documentation-based Pittsburgh Knee Rule classification and radiographic findings Percentages in the fracture and no-fracture columns are calculated within each documentation category. Percentages in the Total column represent the proportion of the overall cohort. PKR: Pittsburgh Knee Rule

Classification	Fracture, N (%)	No fracture, N (%)	Total, N (%)
Documented PKR-positive	15 (18.1%)	68 (81.9%)	83 (41.1%)
Indeterminate documentation	4 (5.1%)	74 (94.9%)	78 (38.6%)
PKR not applicable	0 (0%)	41 (100%)	41 (20.3%)
Documented PKR-negative	0	0	0 (0%)
Total	19 (9.4%)	183 (90.6%)	202 (100%)

Re-review of the original "PKR criteria not met" group

The original analysis had grouped 91/202 (45.0%) requests as not demonstrating documented PKR criteria supporting radiography. Detailed re-review demonstrated that this binary classification did not adequately distinguish genuinely negative clinical findings from incomplete documentation.

Of these 91 requests, 61/91 (67.0%) were reclassified as indeterminate because one or more elements required to establish PKR status were not documented. Twenty-eight of 91 (30.8%) involved mechanisms for which the PKR was not applicable, while 2/91 (2.2%) were reclassified as documented PKR-positive. None of the 91 requests contained sufficient information to establish a confidently documented PKR-negative status.

Fracture yield

Acute fractures were identified in 19/202 (9.4%) examinations. Among the 83 documented PKR-positive requests, fractures were identified in 15/83 (18.1%). Four fractures were identified among the 78 requests with indeterminate documentation, corresponding to 4/78 (5.1%). No fractures occurred among the 41 requests classified as PKR not applicable.

Two fracture-associated requests had originally been included within the group classified as not meeting documented PKR criteria. Both involved patients aged between 12 and 50 years and documented a fall mechanism; however, neither request documented whether the patient was able to walk four weight-bearing steps. These cases were therefore reclassified as indeterminate rather than PKR-negative.

The specific fracture types were not retained within the audit dataset and could not be reliably reconstructed retrospectively.

## Discussion

This audit identified substantial variation in the completeness of clinical information documented on knee radiograph requests with reference to the PKR. The principal finding was not that a large proportion of patients definitively failed to satisfy the PKR, but that a substantial proportion of requests did not contain enough documented information to determine PKR status reliably.

This distinction is important because the present study assessed information retrospectively recorded within radiology requests rather than clinicians' real-time clinical assessment or prospective application of the rule. Absence of a documented criterion cannot therefore be assumed to represent absence of the corresponding clinical finding. Similarly, incomplete documentation does not establish that radiography was clinically inappropriate.

A re-review of the original binary classification illustrates this methodological issue. The initial analysis had categorised 91 requests as not meeting the documented PKR criteria. After distinguishing missing documentation from explicitly negative findings and considering whether the documented mechanism was appropriate for the application of the PKR, none of these 91 requests represented a confidently documented PKR-negative case. Most were either indeterminate because a required clinical element had not been documented or involved mechanisms outside the intended scope of the rule.

This finding also explains why the diagnostic-performance estimates included in the original analysis were removed. Calculation of sensitivity, specificity, and predictive values requires reliable classification of both positive and negative cases. In this retrospective documentation audit, a confidently documented PKR-negative cohort could not be established without assuming that undocumented clinical findings were absent. Such calculations would therefore risk measuring the completeness of request documentation rather than the diagnostic performance of the PKR itself.

Mechanism of injury was also an important consideration. The PKR was developed for patients presenting following a fall or blunt trauma [2,3]. Isolated twisting, hyperextension, and other non-blunt mechanisms should therefore not automatically be interpreted as negative PKR presentations. Such requests were classified separately as PKR not applicable in the revised analysis.

The overall fracture yield in this audit was 19/202 (9.4%), supporting previous observations that only a minority of knee radiographs following acute injury demonstrate fracture. Konan et al. reported that 101/106 (95.3%) patients in their prospective cohort underwent knee radiography, while fractures were identified in 5/106 (4.7%) [1]. In their study, the authors estimated that the application of the PKR could have avoided approximately 30% of radiographs without missing a fracture [1]. This estimated reduction was reported by Konan et al. and should not be interpreted as an estimate derived from the present cohort.

The original validation study and subsequent multicentre evaluation reported high sensitivity for the PKR [2,3]. Direct numerical comparison with the present audit is limited, however, because those investigations involved systematic prospective assessment of the relevant clinical variables. In contrast, the present study retrospectively assessed information recorded within routine radiology requests. The purpose of this audit was therefore to evaluate documentation quality and requesting practice rather than to validate the diagnostic performance of the PKR.

The findings remain clinically relevant because the RCR AuditLive standard specifically recommends that relevant imaging requests contain sufficient clinical information to permit assessment with reference to the PKR [4]. In the present audit, 78/202 (38.6%) requests were indeterminate because the available documentation was insufficient to establish PKR status. Improved documentation of the mechanism of injury and weight-bearing ability would allow imaging requests to be assessed more reliably and would improve communication between requesting clinicians and the radiology service.

The fracture distribution should also be interpreted cautiously. Fifteen of 19 (78.9%) fractures occurred in requests classified as documented PKR-positive, while four occurred in the indeterminate group. The presence of fractures among indeterminate requests does not represent failure of the PKR because the necessary clinical information was not available retrospectively to determine the true rule status of these patients.

Following completion of the first audit cycle, an educational poster was displayed within the Accident and Emergency department. The intervention was intended to increase awareness of the PKR and prompt documentation of the mechanism of injury, relevant age criteria, and four-step weight-bearing ability. A second audit cycle is planned to determine whether this intervention results in measurable improvement in request documentation.

Limitations

Several limitations should be recognised. First, this was a retrospective, single-centre clinical audit and therefore depended on the completeness and accuracy of information recorded within radiology requests. Clinical findings that may have been assessed but not documented could not be independently verified.

Second, this limitation directly affected classification. A documented PKR-negative result required explicit evidence of an applicable mechanism, age between 12 and 50 years, and ability to walk four weight-bearing steps. No request contained all of the information required to establish such a negative group confidently.

Third, mechanisms outside a fall or blunt trauma fall outside the intended scope of the original PKR and were therefore analysed separately rather than being classified as negative.

Fourth, although 300 records were initially identified and 95 were excluded during eligibility screening, the historical screening record did not consistently retain a discrete exclusion reason for every excluded examination. Individual exclusion categories could therefore not be reconstructed reliably for all excluded records.

Fifth, detailed fracture morphology was not retained within the final audit dataset. Consequently, the specific fracture types for the fracture-associated cases could not be reported retrospectively.

Finally, only 19 fractures were identified, limiting conclusions regarding differences in fracture yield between documentation categories.

## Conclusions

This audit identified variation in the adequacy of clinical information documented on knee radiograph requests with reference to the PKR. More than one-third of requests were indeterminate because the documented information was insufficient to establish PKR status. These findings reflect the information available within radiology requests rather than confirmed real-time application of the PKR, and absence of documented evidence should not be interpreted as evidence that the relevant clinical finding was absent or that imaging was inappropriate.

More consistent documentation of the mechanism of injury, patient age, and ability to walk four weight-bearing steps would permit more reliable assessment of knee radiograph requests against established clinical decision rules. An educational intervention has subsequently been introduced within the Accident and Emergency department, and a second audit cycle is planned to assess whether this improves PKR-related documentation and supports more evidence-based imaging-requesting practice.
